# Supplementary figures and images for: Recognition of a highly conserved glycoprotein B epitope by a bivalent antibody neutralizing HCMV at a post-attachment step
Source: PLoS Pathog. 2020 Aug 3;16(8):e1008736. doi: 10.1371/journal.ppat.1008736 (PMC7425986; doi:10.1371/journal.ppat.1008736)

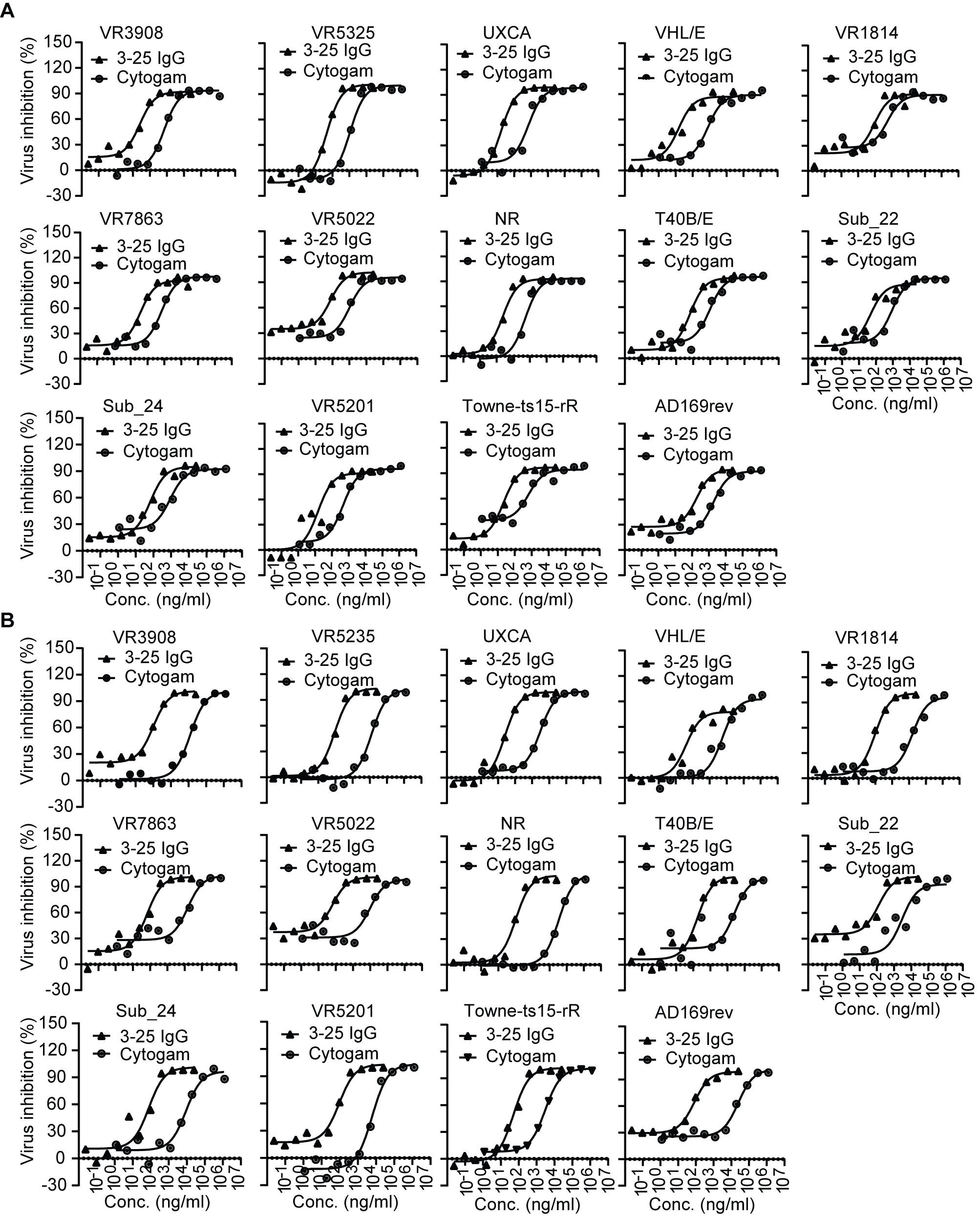

Supplement: S1 Fig — Twelve clinical HCMV isolates and two laboratory-adapted HCMV strains were used for neutralization assays in (A) ARPE-19 cells and (B) MRC-5 cells. The IC50 was calculated by non-linear fit of the percentage of virus inhibition vs. concentration (ng/mL). (TIF) [file ppat.1008736.s001.tif]

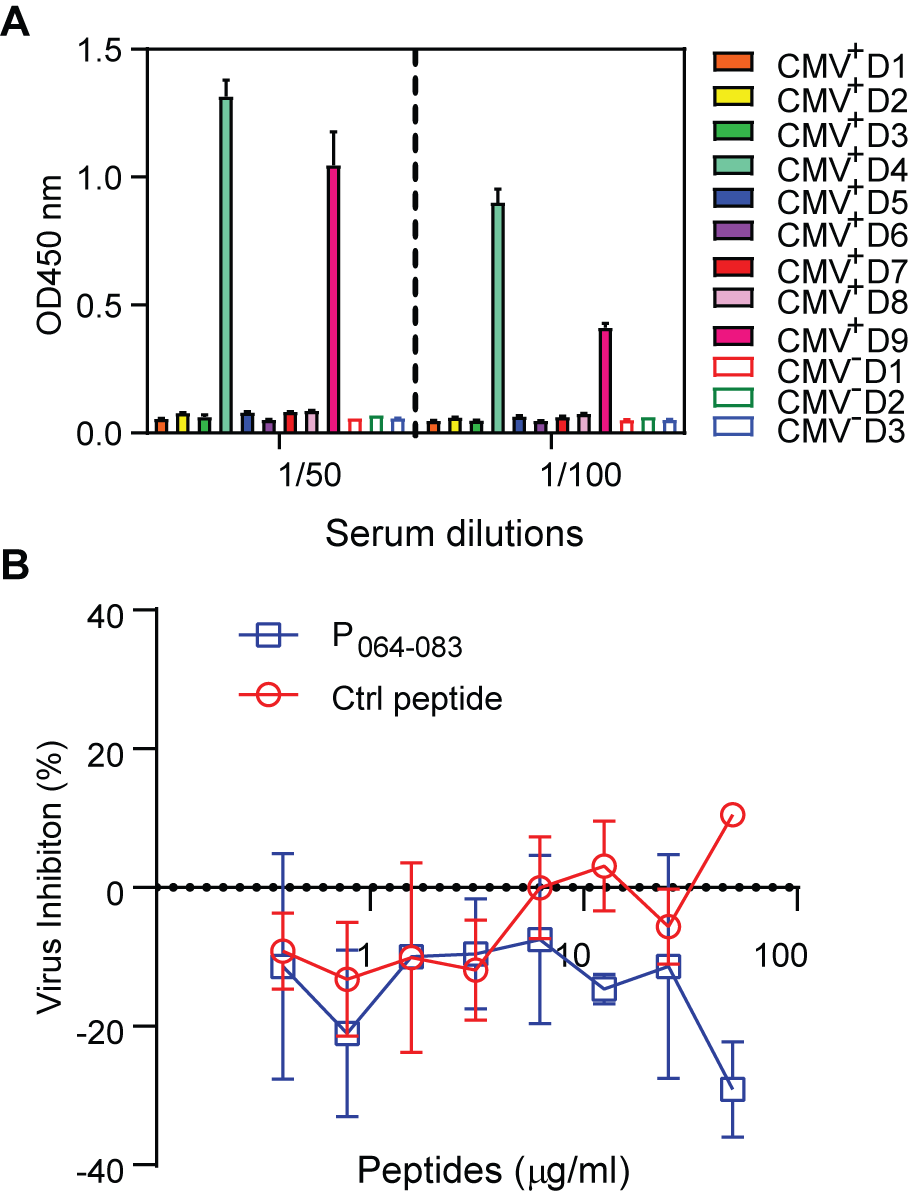

Supplement: S2 Fig — (A) Detection of 3–25 epitope-peptide-specific antibody responses in serum samples of 9 HCMV seropositive and 3 HCMV seronegative individuals by ELISA assay. (B) 3–25 epitope peptide gB P064-083 or a control peptide at different concentrations were pre-incubated with ARPE-19 cells for 1 h before AD169rev-GFP infection. Virus infection as indicated by GFP was quantified using a C.T.L. Immunospot analyzer. (TIF) [file ppat.1008736.s002.tif]

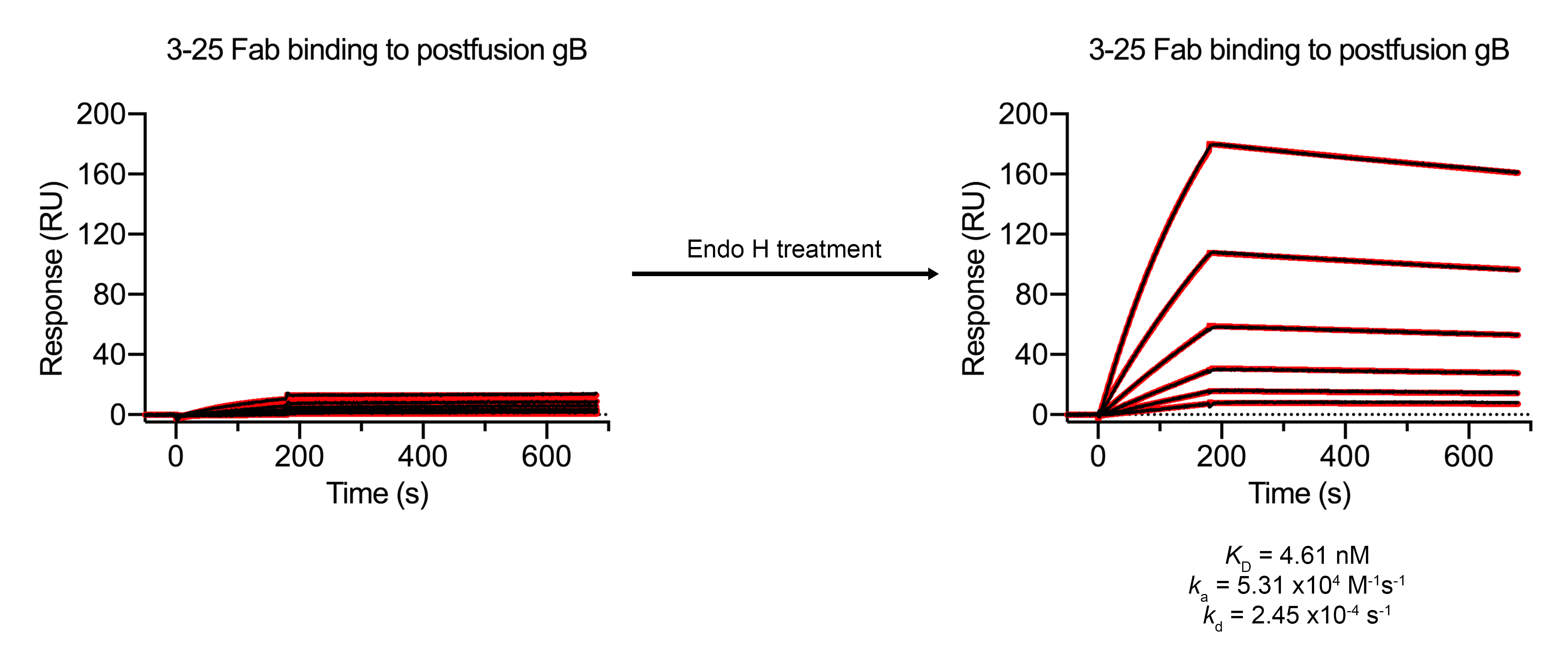

Supplement: S3 Fig — Sensorgrams showing the binding of 3–25 Fab to glycosylated postfusion gB (left) or Endoglycosidase H (Endo H)-treated postfusion gB (right) are shown. SPR response curves are shown as black lines and the fits used to calculate binding kinetics are shown as red lines. (TIF) [file ppat.1008736.s003.tif]

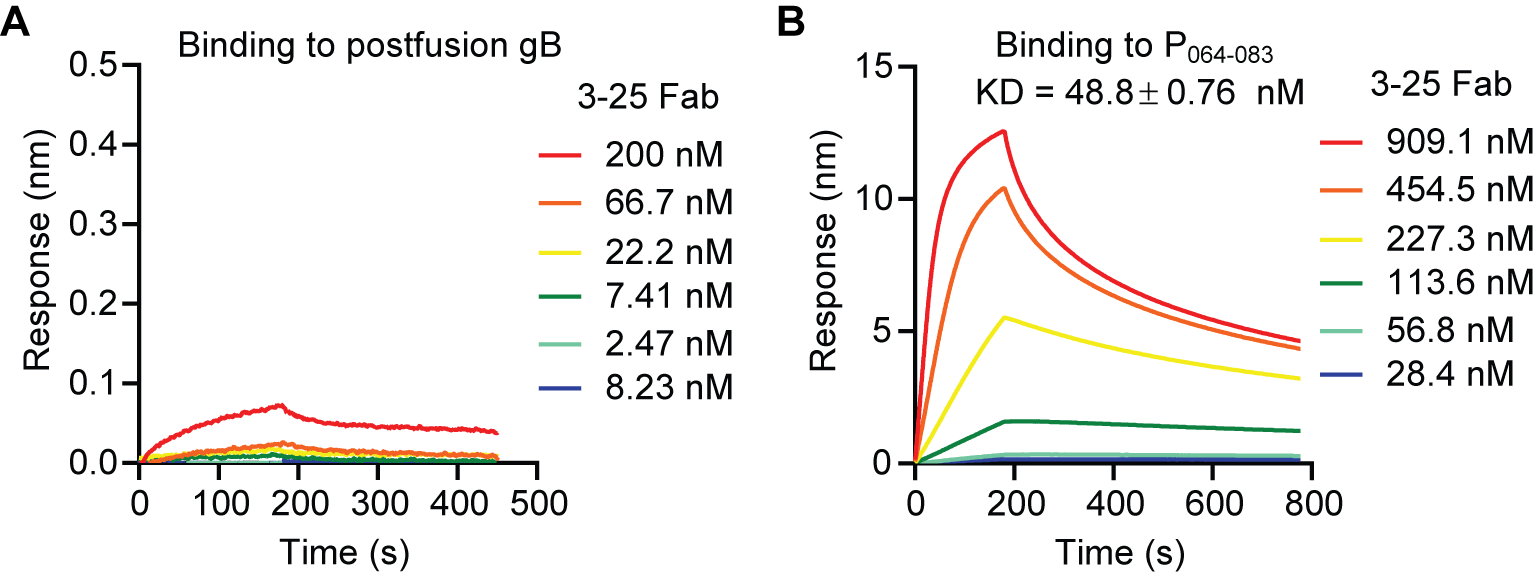

Supplement: S4 Fig — (A) The binding of 3–25 Fab to postfusion gB that was captured on Ni-NTA biosensors and (B) the binding of 3–25 Fab to biotinylated epitope peptide gB P064-083 that was captured on streptavidin biosensors were determined by bio-layer interferometry (BLI) assay. (TIF) [file ppat.1008736.s004.tif]

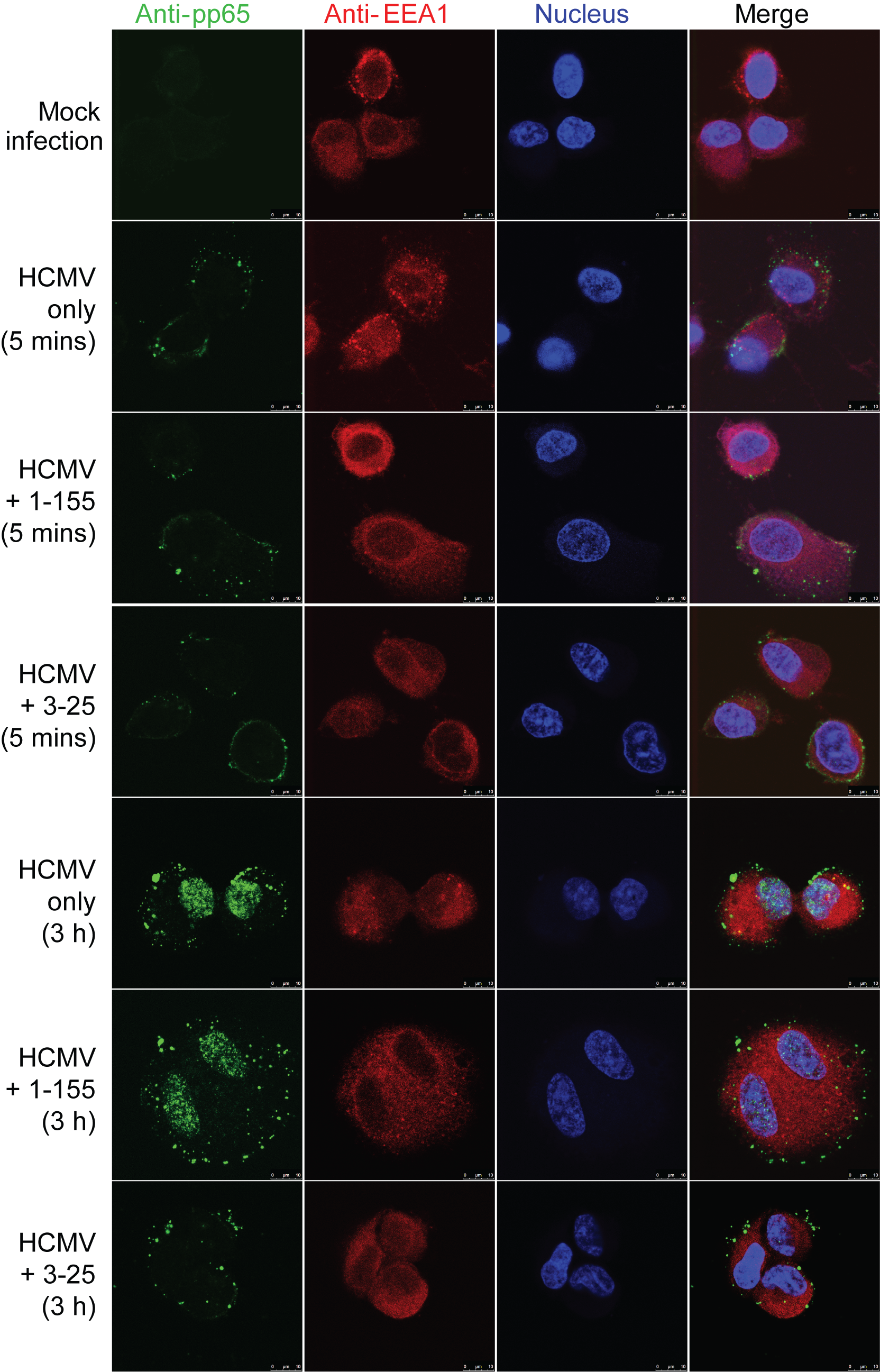

Supplement: S5 Fig — ARPE-19 cells grown in chamber slides were attached with AD169rev at a MOI = 10. After removing unbound virus, 10 μg/mL of 3–25 and 1–155 were added to the cells and then cultured at 37°C for 5 min or 3 h. The cells were fixed, permeabilized, blocked, and double stained with mouse anti-pp65 and rabbit anti-EEA1 antibodies, and corresponding fluorescently labelled secondary antibodies. Nuclei were stained with To-pro-3 (blue). Bar = 10 μm. (TIF) [file ppat.1008736.s005.tif]
