# Supplementary material for: Recognition of a highly conserved glycoprotein B epitope by a bivalent antibody neutralizing HCMV at a post-attachment step
Source: PLoS Pathog. 2020 Aug 3;16(8):e1008736. doi: 10.1371/journal.ppat.1008736 (PMC7425986; doi:10.1371/journal.ppat.1008736)
Supplement: S1 Table — (DOCX) [file ppat.1008736.s007.docx]

**S1 Table.** IC50 of 3-25 and CytoGam against infection of a panel of HCMV isolates in ARPE-19 cells and MRC-5 cells.

| **Viral strains** | **ARPE-19 cells** | | **MRC-5 cells** | |
| --- | --- | --- | --- | --- |
|  | **3-25 (ng/mL)** | **Cytogam (ng/mL)** | **3-25 (ng/mL)** | **Cytogam (ng/mL)** |
| **VR3908** | 25.37±7.0 | 684.5±152.1 | 99.03±28.39 | 12272 ±2533 |
| **VR5325** | 45.29±9.58 | 1070±268.1 | 91.61±15.49 | 12541±3121 |
| **UXCA** | 16.48±1.72 | 699.6±276.1 | 24.77±1.39 | 3038±3.48 |
| **VHL/E** | 15.0±7.38 | 786.7±152.4 | 40.97±16.13 | 5510±1979 |
| **VR1814** | 95.19±52.04 | 569.9±229.3 | 95.6±20.40 | 15890±4701 |
| **VR7863** | 36.71±9.43 | 688.7±147.7 | 66.39±30.01 | 15063±7317 |
| **VR5022** | 74.94±19.25 | 1016±298.8 | 66.05±21.48 | 9579±4528 |
| **NR** | 23.47±5.88 | 521.8±187.6 | 105.7±34.07 | 34296±4578 |
| **T40B/E** | 65.19±15.63 | 936.1±306.3 | 175±66.42 | 25505±5565 |
| **Sub 22** | 45.95±25.09 | 873.9±311.4 | 124.2±48.36 | 2904±1848 |
| **Sub 24** | 65.39±13.16 | 874.5±363.8 | 77.06±40.8 | 9123±3530 |
| **VR5201** | 14.88±9.67 | 457±77.89 | 93.7±23.84 | 6029±1321 |
| **Towne-ts15-rR** | 21.39±3.56 | 634.7±247.9 | 50.6±9.85 | 3807.6±237.8 |
| **AD169rev** | 188.3±56.90 | 1848±443.4 | 80.6±11.76 | 24677±5313 |
