# Supplementary material for: Recognition of a highly conserved glycoprotein B epitope by a bivalent antibody neutralizing HCMV at a post-attachment step
Source: PLoS Pathog. 2020 Aug 3;16(8):e1008736. doi: 10.1371/journal.ppat.1008736 (PMC7425986; doi:10.1371/journal.ppat.1008736)
Supplement: S2 Table — (DOCX) [file ppat.1008736.s008.docx]

**S2 Table.** The gB sequences of clinical isolates used in neutralization assays.

| **Viral strains** | **Sequences of gB (32-88 aa)** |
| --- | --- |
| AD169 | TSSTHNGSHTSRTTSAQTRSVYSQHVTSSEAVSHRANETIYNTTLKYGDVVGVNTTK |
| VR1814 | TSSAHNGSHTSRTTSAQTRSVSSQHVTSSEAVSHRANETIYNTTLKYGDVVGVNTTK |
| UXCA | -S-THNGNHTSHTTSAQTRSISSQRVTSSEAVSHRANETIYNTTLKYGDVVGVNTTK |
| VR3908 | SATHSHHSSHTTSAAHSRSGSVSQRVTSSQTVSHGVNETIYNTTLKYGDVVGVNTTK |
| VR7863 | SATHSHHSSHTTSAAHSRSGSVSQRVTSSQTVSHGVNETIYNTTLKYGDVVGVNTTK |
| VR5235 | SATHSHHSSHTTSAAHSRSGSVSQRVTSSQTVSHGVNETIYNTTLKYGDVVGVNTTK |
| VR5201 | SATHSHHSSHTTSAAHSRSGSVSQRVTSSQTVSHGVNETIYNTTLKYGDVVGVNTTK |
| VHL/E | SATHSHHSSHTTSAAHSRSGSVSQRVTSSQTVSHGVNETIYNTTLKYGDVVGVNTTK |
| NR | SATHSHHSSHTTSAAHSRSGSVSQRVTSSQTVSHGVNETIYNTTLKYGDVVGVNTTK |
| T40B/E | SATHSHHSSRTTSAAHSRSGSVSQRVTSSQTVSHGVNETIYNTTLKYGDVVGVNTTK |
| Sub 22 | SATHSHHSSHTTSAAHSRSGSVSQRVTFSQTVSHGVNETIYNTTLKYGDVVGVNTTK |
| Sub 24 | SATHSHHSSHTTSAAHSRSGSVSQRVTFSQTVSHGVNETIYNTTLKYGDVVGVNTTK |
| VR5022 | SATHSHHSSHTTSAAHSRSGSVSQRVTFSQTVSHGVNETIYNTTLKYGDVVGVNTTK |

Core epitope of 3-25 is underlined. Predicted asparagine *N*-glycosylation sites are colored red.
