# Supplementary material for: Recognition of a highly conserved glycoprotein B epitope by a bivalent antibody neutralizing HCMV at a post-attachment step
Source: PLoS Pathog. 2020 Aug 3;16(8):e1008736. doi: 10.1371/journal.ppat.1008736 (PMC7425986; doi:10.1371/journal.ppat.1008736)
Supplement: S3 Table — (DOCX) [file ppat.1008736.s009.docx]

**S3 Table.** X-ray crystallographic data collection and refinement statistics.

|  | **3-25 Fab + gB-p17** |
| --- | --- |
| **PDB ID** | **6UOE** |
| **Data collection** |  |
| Space group | *C*2 |
| Cell dimensions |  |
| *a, b, c* (Å) | 188.9, 67.8, 84.5 |
| α, β, 𝛾 (°) | 90.0, 133.0, 90.0 |
| Wavelength (Å) | 1.54178 |
| Resolution (Å) | 33.32-1.80 (1.86-1.80) |
| Unique reflections | 41,589 (2,525) |
| R_merge_ | 0.051 (0.197) |
| R_pim_ | 0.050 (0.194) |
| I/σI | 10.2 (3.0) |
| CC_1/2_ | 0.995 (0.910) |
| Completeness (%) | 92.1 (95.5) |
| Redundancy | 2.5 (2.3) |
| Wilson *B*-factors (Å^2^) | 18.6 |
|  |  |
| **Refinement** |  |
| Resolution | 33.32-1.80 (1.86-1.80) |
| Unique reflections | 41,535 (4,293) |
| *R*_work_/*R*_free_ (%) | 16.0/18.6 |
| No. atoms |  |
| Protein | 3430 |
| Water | 646 |
| Ligand (TRS, PEG) | 15 |
| B-factors (Å^2^) |  |
| Protein | 19.9 |
| Water | 35.2 |
| Ligands (TRS, PEG) | 36.3 |
| R.m.s. deviations |  |
| Bond lengths (Å) | 0.01 |
| Bond angles (°) | 1.14 |
| Ramachandran |  |
| Favored (%) | 99.1 |
| Allowed (%) | 0.9 |
| Outliers (%) | 0.0 |
