# Supplementary material for: Recognition of a highly conserved glycoprotein B epitope by a bivalent antibody neutralizing HCMV at a post-attachment step
Source: PLoS Pathog. 2020 Aug 3;16(8):e1008736. doi: 10.1371/journal.ppat.1008736 (PMC7425986; doi:10.1371/journal.ppat.1008736)
Supplement: Attachment — Submitted filename: Response to reviewers.docx [file ppat.1008736.s010.docx]

Reviewer Comments

**Part I - Summary**

Reviewer #1: This study characterises a monoclonal antibody against gB of HCMV identifying the epitope, demonstrating anti-HCMV activity in vitro argue that it works post binding to neutralise infection and also can limit cell to cell spread in a multi-step growth assay. Finally, they suggest that it works via bi-valent binding although no mechanistic data to support this is divalent binding is required for activity is provided. Overall the experiments are generally well executed and presented fairly although some clarifications are needed in certain areas.

Response: We thank the reviewer for the comments. We have made clarifications as suggested. Please check our point-to-point responses below.

Reviewer #2: This paper by Ye et al. presents a functional analysis of 3-25, a human monoclonal antibody recognizing the HCMV fusion protein gB. The authors show that 3-25 has a broad and potent neutralizing activity against 14 HCMV strains. It binds to a specific epitope within AD-2, which is highly conserved among all known HCMV gB proteins. Binding to this epitope was also confirmed in structural studies with 3D reconstruction. By binding to gB, 3-25 blocks the fusion of the viral envelope with cellular membranes and inhibits gB-dependent cell-to-cell spread. Finally, the authors show that bivalent binding of 3-25 to gB is required for neutralization.

Overall, the data are very clear and convincing, and the paper is well written. This study is the most detailed and comprehensive analysis of an HCMV-neutralizing antibody I have seen in a single paper, and this is also the greatest strength of the study. A weakness is that most of the analyses and key findings are not really novel. Co-crystallizations of gB peptides with neutralizing antibody fragments have been done before, and the requirement of bivalent binding for neutralizing antibodies directed against AD-2 has also been shown before. Nevertheless, the beautiful and comprehensive analysis of a potent gB-neutralizing antibody with great potential for clinical application make this study a valuable contribution to the field.

Response: We appreciate this reviewer for the comments and all of the helpful suggestions for us to improve the manuscript. We removed two sentences from our manuscript to tone down our claim of structural related study.

Reviewer #3: In this manuscript, Ye et al reported the functional, biochemical and structural characterization of an antibody, 3-25, isolated from a HCMV seropositive donor. 3-25 potently neutralizes epithelial and fibroblast cells infection by 14 HCMV strains tested and it appears to require the bivalency in the IgG or (Fab)2 format for activity. Functional analysis further demonstrated that 3-25 does not interfere with HCMV attachment and neutralizes HCMV by preventing the fusion process to occur. Epitope mapping showed that 3-25 targets the conserved site I portion of the antigenic domain 2 (AD2) on HCMV gB. Crystal structure of 3-25 Fab in complex with the gB epitope was determined by X-ray Crystallography at 1.8A resolution. Furthermore, negative-stain EM analysis was used to show the location of the 3-25 binding site on the postfusion conformation of the HCMV gB.

Overall, this manuscript is of adequate quality for publication on PLoS Pathogen. My only major concern is that the AD2 epitope on the prefusion conformation of gB is likely to have a different structural arrangement than on the postfusion conformation, thus the structural data presented here represents an incomplete structural basis for 3-25 neutralization. I note that the authors acknowledge this potential drawback in the discussion.”. Given that the functional characterization (6 figures) outweighs structural data (1 figure), the title should be modified to reflect this.

Response: We appreciate this reviewer’s comments and the constructive suggestions on our manuscript. As suggested here, we changed the title of our manuscript to be “Recognition of a highly conserved glycoprotein B epitope by a bivalent antibody neutralizing HCMV at a post-attachment step”. The running title was modified to be “Characterization of a HCMV gB-specific neutralizing antibody.” The keywords were changed accordingly to: “Glycoprotein B, antigenic domain II, crystal structure, neutralizing antibody”. Please see the revised manuscript.

**Part II – Major Issues: Key Experiments Required for Acceptance**

Reviewer #1: I have no major criticisms that absolutely need addressing experimentally.

Responses: Thanks.

Reviewer #2: 1. The authors claim to have resolved the neutralizing mechanism of 3-25, as stated explicitly in the running title, keywords, abstract, and elsewhere in the manuscript. However, the underlying molecular mechanism does not seem to be fully explained by the data. Of course, every neutralizing anti-gB antibody must bind to gB and inhibit its main function, i.e. fusion. But how does it inhibit fusion? Does it prevent interaction with gH/gL, membrane insertion, or the conformational switch? A previous paper on a gB-neutralizing antibody used structural data to propose that neutralization occurs by blocking the pre- to postfusion transition of gB (Spindler et al., PLoS Pathog. 2014). The authors need to explain how 3-25 prevents fusion or at least present a plausible model.

Response: We appreciate the reviewer’s comments. As described above in our response to the summary by reviewer #3, we modified the title, running title and keywords of our manuscript. We also added explanations regarding the neutralizing mechanism of 3-25 to the discussion section of our revised manuscript (See lines 366-386). Those details are also summarized below.

The prefusion gB structure of herpesvirus (including HCMV) is still not available, which leaves a major gap in our knowledge about the mechanism of herpesviruses membrane fusion. The postfusion gB structure of HCMV resembles the postfusion structure of HSV-1 and EBV homologues [1]. The epitope of 3-25 is located at the N-terminal region of HCMV gB. Due to high flexibility, the N-terminal region is unresolved in all currently available gB structures [1]. Similar to HCMV, the N-terminal region of HSV-1 gB is also targeted by neutralizing antibodies, but the essential function of this region for virus entry is still unknown [2]. Results of a study suggest the domain II of HSV-1 gB is involved in interaction with gH/gL and is essential for viral membrane fusion [3]. Two putative hydrophobic fusion loops are located at domain I of HCMV gB [4]. The 3-25 epitope is located at the crown end of postfusion gB, which is distant from domain II that is in the center of gB and the fusion loops containing domain I that is at the opposite base end of gB. Thus, it is unlikely that 3-25 can directly inhibit membrane insertion of fusion loops or gB interaction with gH/gL. We propose that the 3-25 antibody binds the N-terminal region on both prefusion gB and postfusion gB. Bivalent binding of 3-25 IgG cross-links adjacent gB subunits to prevent the concerted gB conformational change that is required for viral membrane fusion. A prefusion gB structure, currently unavailable, would reveal the mechanism of gB-mediated membrane fusion and provide us a basis for forming a hypotheses about the precise neutralization mechanism of the gB N-terminal specific antibodies.

2. The authors argue that 3-25 prevents envelope fusion with endosomal membranes. As a result, pp65 is trapped in cytoplasmic dots (presumably endosomes) and does not translocate to the nucleus. In Figures 5 and S4, pp65 does not co-localize with EEA1, a marker for early endosomes. However, the EEA1 staining is quite unusual (why are nuclei EEA1 positive?), raising doubts on the specificity of the EEA1 antibody. More convincing data with a better endosome-specific antibody is needed.

Response: We thank the reviewer for these comments. We used an anti-EEA1 antibody (#ab2900, Abcam) that has been referenced in over 160 publications in our experiments. The slightly EEA1 staining in nuclei may be caused by over exposure. We repeated the experiments in Fig 5 and S4 Fig with optimized exposure time and got improved EEA1 staining. The new data were shown below as **Fig R1** and **Fig R2**. We replaced the old Fig 5 and S4 Fig with these new figures.

Cellular entry of HCMV can happen via different routes. HCMV enters fibroblast cells through direct fusion with plasma membrane or micropinocytosis [5-9]. HCMV entry into epithelial and endothelial cells relies on endocytosis and low-pH fusion [8,10,11]. We showed that 3-25 antibody efficiently neutralized HCMV infection in both MRC-5 cells and ARPE-19 cells. 3-25 should inhibit HCMV envelope fusion with both plasma membrane and endosomal membrane. We observed no obvious co-localization of trapped virions and the early endosome marker EEA1 in either MRC-5 or ARPE-19 cells. The reasons may include: 1) HCMV does not use the endocytosis pathway as a main route to enter the cells and 2) the co-localization of HCMV virions with early endosomes is temporary and needs to be captured at time points other than those we used.

**Fig R1**. 3-25 arrests internalized HCMV virions around the perinuclear region in MRC-5 cells. MRC-5 cells grown in chamber slides were attached with AD169rev at a MOI=10. After removing unbound virus, 10 µg/mL of 3-25, 1-155 or control IgG was added to the cells and then cultured at 37 °C for 5 min or 3 h. The cells were fixed, permeabilized, blocked, and double stained with mouse anti-pp65 and rabbit anti-EEA1 antibodies, and corresponding fluorescently labelled secondary. Nuclei were stained with To-pro-3 (blue). Bar=10 µm.

**Fig R2**. 3-25 arrests cell-attached virions and inhibits viral membrane fusion in ARPE-19 cells. ARPE-19 cells grown in chamber slides were attached with AD169rev at a MOI=10. After removing unbound virus, 10 µg/mL of 3-25 and 1-155 were added to the cells and then cultured at 37 °C for 5 min or 3 h. The cells were fixed, permeabilized, blocked, and double stained with mouse anti-pp65 and rabbit anti-EEA1 antibodies, and corresponding fluorescently labelled secondary antibodies. Nuclei were stained with To-pro-3 (blue). Bar=10 µm.

3. The authors use AD159rev for most of their experiments. Please explain why this HCMV strain was used, even though it is known to have an unusual behavior: it induces extensive cell-cell fusion in infected epithelial cells (PMID 16051825). The AD169 gB has recently been shown to be hyperfusogenic and mediate rapid entry into MRC5 fibroblasts (PMID 31427511). Could this explain the "surprising" finding described in lines 221-223? Do the authors see the same phenomenon when they use another HCMV strain?

Response: We appreciate the reviewer’s comments. We used the AD169rev strain in most experiments because of its accessibility. The paper cited (PMID 16051825) demonstrated that the UL131-128 locus of HCMV is required for efficient infection of epithelial cells and cell-cell fusion [12]. The authors did mention that repaired AD169 virus leads to extensive membrane fusion and syncytium formation, [12]. However, these phenomena were also observed in other HCMV strains, including clinical isolates [13-15]. The paper cited (PMID 31427511) showed that HCMV activates caspase-2 in infected fibroblast cells depending on the gB protein of AD169, which carries a gB 275D variant that has increased fusogenicity and accelerated viral entry [16]. Thus, the unusual behavior of AD169 strain and its derivate is higher infectivity, in which the hyperfusogenic gB variant plays a role. The AD169 strain and its derivative are therefore more difficult neutralize by gB-specific antibodies than other HCMV strains, but the viral membrane fusion mechanism remains the same. Therefore, AD169rev strain should be representative of other HCMV strains. Our normal neutralization assay showed that 3-25 efficiently neutralized AD169rev together with other 13 HCMV strains (Fig 1 and S1 Table). Although we did not perform post-attachment assay using other HCMV strains, we speculate to see the same phenomenon as that of AD169rev.

As stated above, HCMV enters different cell types via different routes. For example, HCMV may enter fibroblast cells through direct fusion with plasma membrane or micropinocytosis [5-9]. HCMV entry into epithelial and endothelial cells may rely on endocytosis and low-pH [8,10,11]. The hyperfusogenic gB caused rapid cell entry of AD169 strain and its derivative should happen in both ARPE-19 cells and MRC-5 cells. We showed that the viral inhibiting efficacy of 3-25 in post-attachment assay was much higher in ARPE-19 epithelial cells than in MRC-5 fibroblast cells (Fig 4E-H). We propose that the poor virus inhibition at post-attachment step by 3-25 in MRC-5 cells may be related to the virus entry route. We made modifications to mention this in the revised manuscript, as “The discrepancy of virus inhibition by 3-25 in ARPE-19 cells and MRC-5 cells at the post-attachment step may be related to different HCMV entry routes in these two cell types” (see line 229-230).

Reviewer #3: (No Response)

**Part III – Minor Issues: Editorial and Data Presentation Modifications**

Reviewer #1: In Figure 6 the authors investigate cell to cell spread and cite the original gB null paper from Teresa Compton who used classical agarose overlays. I think it is useful to clarify what the authors mean. They are using an AD169 repaired virus so the implication is that it is cell associated not cell free. However, does not a reduction in plaque number also suggest cell free virus under the conditions they are using (no overlay). Have they analysed the amount of cell free versus cell associated virus there are in these cultures? Some comment would be appreciated on the limitations of interpretation.

Response: Thank you for the comments. To clarify, we cited the original gB-null paper to state the validated function of gB in HCMV infection. The AD169 repaired virus (AD169rev) we used produces both cell-associated virus and cell-free virus during culture; production of the cell-associated virus is slightly higher than the cell-free virus [12]. We did not use the classical agarose overlay assay, which specifically determines HCMV cell-to-cell spread. The assay we did use for results shown in Fig 6 determined the overall viral inhibiting effect of 3-25 when added to cell culture at three days post-infection. We made modifications to clarify this: “Glycoprotein B is strictly required for HCMV entry and cell-to-cell spread as demonstrated by a gB-null virus. Here we determined whether 3-25 inhibits post-infection HCMV shedding and spreading” (see lines 251-252).

My reading of the paper is that binding of the antibody to the virion is monovalent, neutralisation on the other hand is bivalent – Here, although they sell it as one of the major findings of the paper, I don’t see how important this is without exploring the mechanism. Is neutralisation bivalent as a result of an increase in affinity of the binding or because bivalent binding prevents conformational changes in gB necessary for fusion? Could the authors comment?

Response: We appreciate these suggestions for clarification. We have added comments addressing these questions to the discussion section of our revised manuscript (See lines 366-386). These comments are briefly summarized below.

Due to the lack of a prefusion gB structure, there is a major gap in knowledge about the mechanism of herpesviruses membrane fusion. The 3-25 epitope is located at the N-terminal region of HCMV gB. The N-terminal region of HSV-1 gB is also targeted by neutralizing antibodies, suggesting an essential function for the gB N-terminal region in viral entry [2]. Due to its high flexibility, the N-terminal region is unresolved in all currently available structures of postfusion gB [1]. However, the location of N-terminal region is distant from the domain of postfusion gB with putative fusion loops [4] or potentially interacting with gH/gL [3]. Thus, the 3-25 antibody inhibits HCMV viral membrane fusion via a distinct mechanism that is associated with the essential function of the gB N-terminal region. A prefusion gB structure, currently unavailable, would reveal the mechanism of gB-mediated membrane fusion and provide a basis for forming a hypothesis about the precise neutralization mechanism of the gB N-terminal region specific antibodies.

The 3-25 Fab, (Fab)_2_, and IgG all showed strong binding to the HCMV virion and gB protein by ELISA assay. However, only the bivalent 3-25 (Fab)_2_ and IgG were able to neutralize HCMV infection. We also observed that the binding affinity of 3-25 Fab to gB was much lower than that of 3-25 IgG, and the binding of 3-25 is sensitive to gB glycosylation. Based on these results, we propose that 3-25 may contribute to HCMV neutralization through 1) increased binding avidity of 3-25 antibody and 2) cross-linking of adjacent gB subunits to prevent the concerted gB conformational change that is required for viral membrane fusion.

They also demonstrate that binding of 3-25 is sensitive to glycosylation (156-158), which may impact neutralisation and efficacy of therapies or vaccines based on anti-AD2 antibodies. However, in spite of incomplete occupancy of the glycosylated form of gB by the Fab, 3-25 Ab neutralises infection. Comment?

Response: We appreciate the question. We showed that the binding of 3-25 Fab to de-glycosylated gB is significantly higher than its binding to glycosylated gB (S3 Fig). However, the 3-25 IgG demonstrated a high binding avidity (1.74 ± 0.06 nM) to glycosylated gB (Fig 4A). Further, 3-25 IgG efficiently neutralized 14 HCMV strains (Fig 1). Thus, in spite of the glycan shield of gB, the epitope is still readily accessible to 3-25. We speculate that HCMV neutralization by 3-25 IgG does not require full saturation of all three of the 3-25 epitopes per gB trimer.

Reviewer #2: 4. Considering that several gB-neutralizing monoclonal antibodies have been characterized before, it would have been great to see a side-by-side comparison of 3-25 and its competitors in order to understand whether or not 3-25 has superior neutralizing activity in vitro and has more potential for clinical application. In the present study, the authors used only one previously described antibody as a control in one set of experiments. While a comprehensive experimental comparison may be beyond the scope of the present study, the authors could at least discuss how their antibody compares to the others, based on the data available.

Response: Thank you for the suggestion. Several side by side neutralization results (including the one shown in the manuscript) showed that the HCMV neutralizing ability of 3-25 is comparable to that of LJP538 developed by Novartis. We added a sentence to discuss this comparison: “Despite recognizing different epitopes, the 3-25 and LJP538 antibodies showed comparable binding and virus neutralizing efficacy” (see lines 287-288).

5. Line 101. the statement "[100–1000](tel:100%E2%80%931000) times lower than the IC50 of CytoGam" is not consistent with the data. 10-200 times lower seems more accurate.

Response: Thank you for the comment. We modified the statement accordingly: “Notably, the IC50 of 3-25 and CytoGam in two cell lines and for all viral strains were 15–188.3 ng/ml and 457–34296 ng/ml (S1 Fig, S1 Table), suggesting that 3-25 is much more potently neutralizing than CytoGam” (see lines 99-101).

6. Line 36. The general statement that HCMV causes asymptomatic infections in otherwise healthy adults is not true. HCMV infections are often mild and oligosymptomatic, sometimes even asymptomatic.

Response: Thank you for the comment. We modified the statement accordingly: “HCMV infection usually causes mild or unnoticeable symptoms in healthy adults” (see line 36-37).

7. Line 233. "with or without antibody for 5 min or 3 h at 37 °C incubator“ – please correct.

Response: We appreciate the suggestion. The mentioned sentence was modified to be “After removing unbound virus, antibodies were added to the cells and cultures were incubated for 5 mins or 3 h at 37 °C” (see lines 236-237).

Reviewer #3: In Fig 2D, it will help readers focus on the changes introduced by highlighting the Ala mutations in boldface. In addition, mutations (for example, Y78A etc) can be added to the right of the bars in the bar graph so it is easier to see the effects of corresponding mutations.

Response: As suggested, we changed the Ala mutations to boldface and added mutations in the bar graph (see Fig 2 in the revised manuscript).

In Fig 3C one Fab was shown to bind to one postfusion gB trimer but in the main text the stoichiometry was described as 3:1. Clarifications need to be added to the figure legend. Alternatively, all three Fabs can be shown in Fig 3C. In Fig 3C, it will also be helpful to label gB, 3-25 Fab and the position of fused membrane.

Response: We appreciate the comment. Due to the high degree of conformational flexibility that we observed in the 3-25 epitope, 3D reconstruction algorithms had a tendency to focus on a single Fab, while the other Fabs were averaged out into noise. However, it is clear from our 2D averages (Fig 3B) that we observed stoichiometric (3:1) 3-25 Fab binding.

As suggested, we labeled gB, 3-25 Fab, and the crown end and fusion loop containing base end of postfusion gB in the Figure (see Fig 3C in the revised manuscript). We also added clarifications to the legend of Fig 3C accordingly: “Three 3D reconstructions of postfusion gB bound by 3-25 Fab are aligned to show the flexibility of the 3-25 epitope. The 3D reconstructions are aligned to one another based on the position of the postfusion gB density and are colored yellow, blue, and red. The crown end of postfusion gB containing 3-25 Fab binding sites and the base end of postfusion gB containing fusion loops are indicated” (see lines 795-799).

In lines 180-181, the authors refer to the interaction between gB Asn73 and Tyr32 of 3-25 CDRL1 as “salt bridge”, this is not correct since salt bridge occurs between oppositely charged amino acids. Hydrogen bonding is more likely (distance and angle between interacting atoms will confirm the interaction type). If hydrogen bonding cannot be concluded based on distance and angle, “polar interaction” can be used here. The interaction between gB Asn73 and 3-25 Tyr32 seems to be in contradiction to the 20-fold higher binding observed when Asn73 is mutated to Ala (Fig 2D).

Response: We appreciate these comments. We replaced “salt bridge” with “hydrogen bond” to describe the interaction between gB Asn73 and Tyr32 of 3-25 CDRL1. Please see line 180 in the revised manuscript.

We added one sentence in the revised manuscript to describe the dramatically increased binding of N73A mutant: “Intriguingly, the N73A substitution in our epitope-mapping experiments resulted in a substantial increase in relative binding potency (Fig 2D), although it should be noted that these experiments were also carried out using peptides that lacked glycosylation” (see lines 183-186). Determining the cause of the increased binding to the N73A mutant would likely require a structure of the mutant peptide in complex with 3-25 Fab. Based on our current structure, the epitope peptide binds to a groove formed by the interface between 3-25 VH and VL (Fig 3D). We speculate that the N73A mutant is able to better fit within this groove than the wildtype peptide, although it should again be noted that Asn73 is an N-linked glycosylation site and these experiments were carried out using synthesized peptides with no glycosylation.

The authors reported a binding affinity of ~2nM between 3-25 IgG and postfusion gB; it will be helpful to obtain affinity of 3-25 Fab. To reduce the avidity effect, it might be helpful to conjugate gB on biosensors and use 3-25 Fab as analyte. The binding affinity cited in the main text is different than the KD shown on Fig 4A.

Response: We appreciate the suggestion. The avidity between 3-25 IgG and postfusion gB in the main text was corrected to be consistent with the KD shown on Fig 4A (see line 197).

In S3 Fig of our manuscript, we used 3-25 Fab as an analyte to determine the binding affinity of 3-25 Fab to postfusion gB and de-glycosylated postfusion gB using a Biacore assay. Our results showed that the binding affinity of 3-25 Fab to glycosylated gB was too low to be calculated. In contrast, the 3-25 Fab showed a high binding affinity (4.61 nM) to de-glycosylated gB.

In addition, we determined the binding affinity of 3-25 Fab to postfusion gB and epitope peptide P_064-083_ by Octet assay. As shown below in **Fig R3**, the 3-25 Fab showed a high binding affinity to the captured epitope peptide but poor binding to the captured postfusion gB. The **Fig R3** was included in the revised manuscript as S4 Fig. Hence the original S4 Fig was changed to S5 Fig. A description was added to the revised manuscript to describe the S4 Fig: “The 3-25 Fab showed a high binding affinity (48.8 ± 0.76 nM) to the captured epitope peptide gB P_064-083_ but poor binding to the captured gB protein.” (See lines 198-199).

**Fig R3**. (**A**) The binding of 3-25 Fab to postfusion gB that was captured on Ni-NTA biosensors and (**B**) the binding of 3-25 Fab to biotinylated epitope peptide gB P_064-083_ that was captured on streptavidin biosensors were determined by bio-layer interferometry (BLI) assay.

**Reference**

1. Burke HG, Heldwein EE (2015) Crystal Structure of the Human Cytomegalovirus Glycoprotein B. PLoS Pathog 11: e1005227.

2. Bender FC, Samanta M, Heldwein EE, de Leon MP, Bilman E, et al. (2007) Antigenic and mutational analyses of herpes simplex virus glycoprotein B reveal four functional regions. J Virol 81: 3827-3841.

3. Atanasiu D, Whitbeck JC, de Leon MP, Lou H, Hannah BP, et al. (2010) Bimolecular complementation defines functional regions of Herpes simplex virus gB that are involved with gH/gL as a necessary step leading to cell fusion. J Virol 84: 3825-3834.

4. Sharma S, Wisner TW, Johnson DC, Heldwein EE (2013) HCMV gB shares structural and functional properties with gB proteins from other herpesviruses. Virology 435: 239-249.

5. Li Q, Fischer E, Cohen JI (2016) Cell Surface THY-1 Contributes to Human Cytomegalovirus Entry via a Macropinocytosis-Like Process. J Virol 90: 9766-9781.

6. Compton T, Nepomuceno RR, Nowlin DM (1992) Human cytomegalovirus penetrates host cells by pH-independent fusion at the cell surface. Virology 191: 387-395.

7. Topilko A, Michelson S (1994) Hyperimmediate entry of human cytomegalovirus virions and dense bodies into human fibroblasts. Res Virol 145: 75-82.

8. Bodaghi B, Slobbe-van Drunen ME, Topilko A, Perret E, Vossen RC, et al. (1999) Entry of human cytomegalovirus into retinal pigment epithelial and endothelial cells by endocytosis. Invest Ophthalmol Vis Sci 40: 2598-2607.

9. Hetzenecker S, Helenius A, Krzyzaniak MA (2016) HCMV Induces Macropinocytosis for Host Cell Entry in Fibroblasts. Traffic 17: 351-368.

10. Ryckman BJ, Jarvis MA, Drummond DD, Nelson JA, Johnson DC (2006) Human cytomegalovirus entry into epithelial and endothelial cells depends on genes UL128 to UL150 and occurs by endocytosis and low-pH fusion. J Virol 80: 710-722.

11. Sinzger C (2008) Entry route of HCMV into endothelial cells. J Clin Virol 41: 174-179.

12. Wang D, Shenk T (2005) Human cytomegalovirus UL131 open reading frame is required for epithelial cell tropism. J Virol 79: 10330-10338.

13. Booth JC, Beesley JE, Stern H (1978) Syncytium formation caused by human cytomegalovirus in human embryonic lung fibroblasts. Arch Virol 57: 143-152.

14. Michelson-Fiske S, Arnoult J, Febvre H (1975) Cytomegalovirus infection of human lung epithelial cells in vitro. Intervirology 5: 354-363.

15. Vonka V, Anisimova E, Macek M (1976) Replication of cytomegalovirus in human epitheloid diploid cell line. Arch Virol 52: 283-296.

16. Tang J, Frascaroli G, Lebbink RJ, Ostermann E, Brune W (2019) Human cytomegalovirus glycoprotein B variants affect viral entry, cell fusion, and genome stability. Proc Natl Acad Sci U S A 116: 18021-18030.
